# Cytomegalovirus Infection during Pregnancy and Its Impact on the Intrauterine Fetal Development – Case Report

**DOI:** 10.3889/oamjms.2016.078

**Published:** 2016-08-08

**Authors:** Mariya Angelova, Emil Kovachev, Nikolai Todorov

**Affiliations:** 1 *Department of Obstetrics and Gynecology, Trakia University, Faculty of Medicine, Stara Zagora, Bulgaria*; 2 *Department of Obstetrics and Gynecology, Paraskev Stoyanov Medical University, Varna, Bulgaria*

**Keywords:** Cytomegalovirus infection, pregnancy, a case report

## Abstract

**AIM:**

The aim of this publication is to present a case of CMV infection during pregnancy, with clinical manifestations of the development of microcephaly and simultaneous dilatation of the 3rd and 4th brain ventricle at 23 weeks gestation. This article discusses the role of ultrasound screening in the second trimester of pregnancy.

**CASE PRESENTATION:**

We present the case of a 25-year-old woman with the initials S.K. in her second pregnancy that came to our antenatal Consulting Centre. The first screening for blood count, blood group, biochemistry and serology showed results within the reference range. The patient came for a second comprehensive biochemical screening at 17 – 18 weeks gestation. The results showed the low genetic risk of congenital anomalies. Fetal morphology of the fetus was normal. S.K. came again for consultation at 22 weeks gestation in connection with the admittance of her first 3-year-old child to the hospital because of pneumonia. Serological tests of the child had shown elevated CMV titer - specific IgM. Then we made new serological tests of the patient and the results have shown that the patient was most likely infected by CMV primarily in the first trimester of pregnancy. After consulting about the risk of transmission of CMV to the fetus, the woman chose monthly ultrasound scans and refused amniocentesis. At 36 weeks gestation, in addition to the microcephaly already established, enlargement of the IV brain ventricle at the expense of underdevelopment of the cerebellum was noticed. Also, 2nd to 3rd stage of placenta maturity and low quantity of amniotic fluid was established. A male fetus of weight 2,890 g and height 50 cm was delivered. The fetus was with skin petechiae and hepatosplenomegaly. Neurological examination showed no abnormalities.

**CONCLUSIONS:**

In the described case the time interval between infection and ultrasonic manifestations is more than 17 weeks. The long interval between infection and occurrence of ultrasound markers can be a good prediction sign, as it may reflect less aggressive viral infection than present in cases where similar ultrasound findings were obtained shortly after infection of the mother.

## Introduction

The cytomegalovirus infection is caused by a DNA virus of the viral family known as herpes viruses. Presently, CMV can be encountered in every fourth woman in childbirth age [1]. Once in the body, the virus remains for a lifetime. Usually, the cytomegalovirus “sleeps” in the body and does not cause any damages [2]. However, when the immunity drops, in cases of frequent colds, stress situations and pregnancy, the virus becomes active [3]. It is important to note that infecting the fetus or the newborn infant can happen only with active virus infection. During pregnancy, the virus attacks the nervous system of the fetus which is connected with early miscarriages or with the development of a number of defects in the newborn infants, whereas 20-30% of them die [4].

The low pathogenicity of the virus in the adult human body is sharply contrasting with the impact on the fetus which can cause serious damages. The harm for the fetus is even more serious when the mother has recently had a primary infection and has not developed any specific CMV antibodies. The clinical forms of fetal damages are: congenital cytomegalic infection acquired perinatal infection and acquired postnatal infection [5].

The focus of this publication is a case of CMV infection during pregnancy which was clinically expressed with the development of microcephaly and simultaneous dilatation of the 3^-rd^ and 4^-th^ cerebral ventricle in the 23 g.w. This paper presents a discussion on the role of the ultrasound testing in the second trimester of pregnancy.

## Case Presentation

The following is a description of the case of a 25-year-old woman with the initials S.K. in her second pregnancy who came to our antenatal consulting centre.

The first screening for blood count, blood group, biochemistry (ALAT, ASAT, creatine, urea, blood sugar) showed results within the reference range. Hbs Ag, HIV, Wasserman, IgG, IgM (toxoplasmosis), IgG, IgM (rubella), IgG, IgM (CMV) were negative.

At that time the woman was at 6 – 7 weeks gestation. Biochemical screening generally is done at 12 – 13 weeks gestation showed results within the reference range (low risk of development of a baby with an abnormality, low risk of developing preeclampsia in the second half of pregnancy and low risk of premature birth).

The patient came for a second comprehensive biochemical screening at 17 – 18 weeks gestation. The results showed the low genetic risk of congenital anomalies. Fetal morphology of the fetus was normal.

S.K. came again for consultation at 22 weeks gestation in connection with the admittance of her first 3-year-old child to the hospital because of pneumonia. Serological tests of the child had shown elevated CMV titer - specific IgM.

Then we made new serological tests of the patient and the results were as follows: CMV – specific IgG – 98; CMV – specific IgM – 2.1. It was then understood that the patient was most likely infected by CMV primarily in the first trimester of pregnancy. After consulting about the risk of transmission of CMV to the fetus the woman chose monthly ultrasound scans and refused amniocentesis.

**Figure 1:**
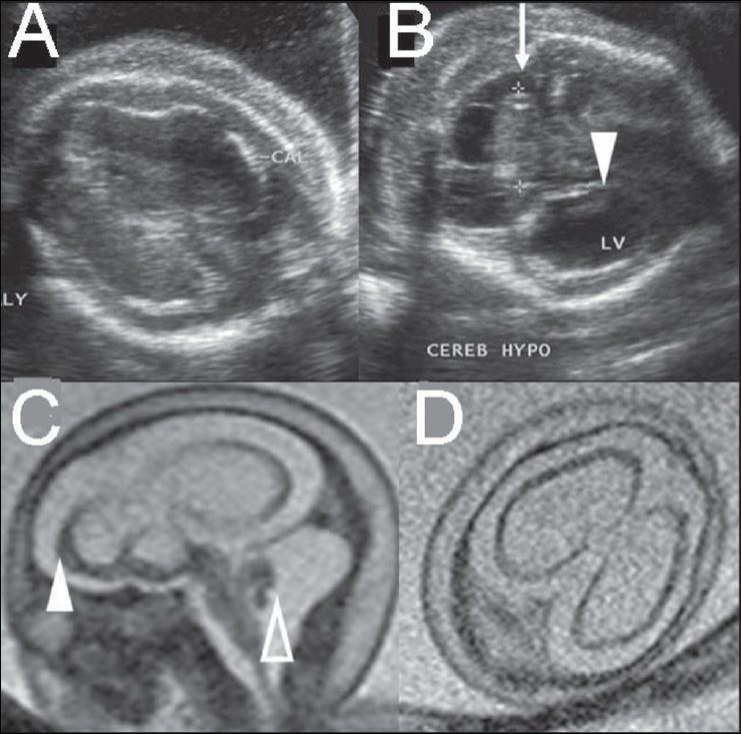
Fetal brain ultrasound scan at 24 weeks gestation

The results of all ultrasound scans were normal until that time. At 24 weeks gestation fetal brain ultrasound scan showed thalamic calcifications bilaterally, hyperechogenic foci in the right side of the ventricular wall, asymmetric ventriculomegaly > 10 mm (Fig. 1).

Similar changes were imaged at 28 weeks gestation and also cerebellar developmental delay was noticed. Also, hepatomegaly was found at otherwise eutrpophically developing a fetus.

After that patient, S.K. consented to do of amniocentesis as changes were already verified by ultrasound. Amniotic fluid analysis detected that the fetus was positive for CMV (CMV – specific IgM titer was 68), thrombocytopenia – 46 g/l. Levels of fetal liver enzymes were normal. Following consultation on the possible consequences of CMV infection of the fetus, the parents chose to continue with the pregnancy.

In the next ultrasound scanning to follow at 32 weeks gestation not only cerebellar developmental delay was noticed, but also of the head as a whole (microcephaly).

**Figure 2:**
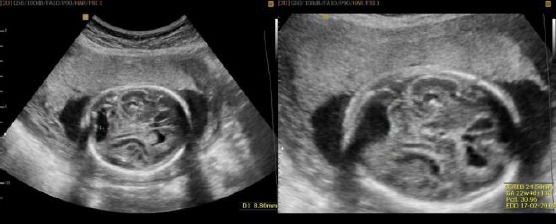
Fetal brain ultrasound scan at 36 weeks gestation

At 36 weeks gestation, in addition to the microcephaly already established, enlargement of the IV brain ventricle at the expense of underdevelopment of the cerebellum was noticed. Also, 2nd to 3rd stage of placenta maturity and low quantity of amniotic fluid was established (Fig. 2).

**Figure 3:**
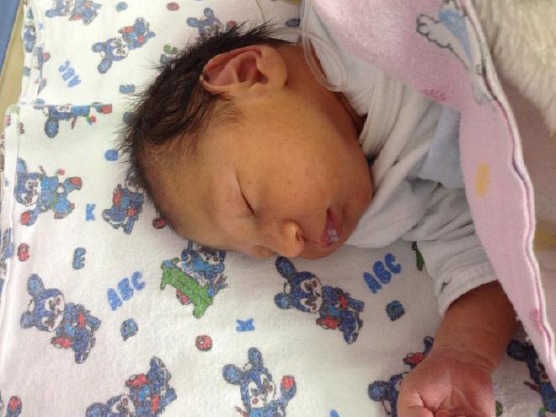
A male fetus of weight 2,890 g and height 50 cm was delivered. The fetus was with skin petechiae and hepatosplenomegaly

The patient was admitted to an obstetrics and gynaecology clinic for active monitoring. Cardiotocography was done on a daily basis. Upon the occurrence of two consecutive non-reactive NST and presence of late decelerations, a decision for delivery was taken. Because of the relatively immature fetus and low pelvic score a caesarian section delivery was done. A male fetus of weight 2,890 g and height 50 cm was delivered. The fetus was with skin petechiae and hepatosplenomegaly. Neurological examination showed no abnormalities. Thrombocytopenia was confirmed (Hg 66 g/l). The newborn baby was treated with Acyclovir i.v. – 5 mg / g / 12 h for 6 weeks (Fig 3).

Presently, the baby is six-month-old, of normal neurological status and preserved hearing.

## Discussion

CMV is the most common congenital viral infection and is the leading infectious cause of sensory deafness and cerebral mental retardation. The estimated risk of vertical transmission in the first trimester of pregnancy is 36-45%. Ten % of infected newborn babies are symptomatic at birth and 15 % of asymptomatic newborn babies display developmental disorders later [6].

In the described case the time interval between infection and ultrasonic manifestations is more than 17 weeks. The long interval between infection and occurrence of ultrasound markers can be a good prediction sign, as it may reflect less aggressive viral infection than present in cases where similar ultrasound findings were obtained shortly after infection of the mother.

This case shows that when there is suspicion of CMV and patients refuse invasive diagnostics (amniocentesis, cordocentesis), monthly ultrasound examinations are of exceptional crucial importance.

According to world statistical data, more than 80% of the population is infected with the virus. Presently, CMV is isolated in every fourth woman of childbearing age. Once this virus enters the body, it remains there for life [7].

Normally, the cytomegalovirus “sleeps” in the body and causes no harm. At fall of immunity, frequent colds, stress and pregnancy, the virus becomes active. It is important to note that infection of the fetus or neonate can take place only in an active viral infection. Only the test by DNA analysis clearly demonstrates active viral infection.

The most serious complication caused by active cytomegalovirus infection during pregnancy is an infection of the fetus or neonate. In pregnancy, the virus affects the nervous system of the fetus which attributes to early miscarriage or development of a number of defects in neonate so that 20-30 % of them die [8].

Unfortunately, in practice congenital cytomegalovirus infection is not susceptible to treatment and therefore taking of preventive measures is required. In pregnant women, it is advisable to monitor and control the activity of the virus by screening in each trimester [9].

In conclusion, the overcoming of the CMV infection is possible through the administration of antiviral drugs. The purpose of such drugs is to suppress viral division (propagation) and to facilitate the passage of the virus from active phase to latent phase of viral rest, in which the virus is not able to infect and harm the body organs.
